# L-Tree: A Local-Area-Learning-Based Tree Induction Algorithm for Image Classification

**DOI:** 10.3390/s18010306

**Published:** 2018-01-20

**Authors:** Jaesung Choi, Eungyeol Song, Sangyoun Lee

**Affiliations:** Department of Electrical and Electronic Engineering, Yonsei University, Seoul 03722, Korea; ciyciyciy@yonsei.ac.kr (J.C.); wp2001@yonsei.ac.kr (E.S.)

**Keywords:** decision tree, ensemble tree, image classification, self-organizing map

## Abstract

The decision tree is one of the most effective tools for deriving meaningful outcomes from image data acquired from the visual sensors. Owing to its reliability, superior generalization abilities, and easy implementation, the tree model has been widely used in various applications. However, in image classification problems, conventional tree methods use only a few sparse attributes as the splitting criterion. Consequently, they suffer from several drawbacks in terms of performance and environmental sensitivity. To overcome these limitations, this paper introduces a new tree induction algorithm that classifies images on the basis of local area learning. To train our predictive model, we extract a random local area within the image and use it as a feature for classification. In addition, the self-organizing map, which is a clustering technique, is used for node learning. We also adopt a random sampled optimization technique to search for the optimal node. Finally, each trained node stores the weights that represent the training data and class probabilities. Thus, a recursively trained tree classifies the data hierarchically based on the local similarity at each node. The proposed tree is a type of predictive model that offers benefits in terms of image’s semantic energy conservation compared with conventional tree methods. Consequently, it exhibits improved performance under various conditions, such as noise and illumination changes. Moreover, the proposed algorithm can improve the generalization ability owing to its randomness. In addition, it can be easily applied to ensemble techniques. To evaluate the performance of the proposed algorithm, we perform quantitative and qualitative comparisons with various tree-based methods using four image datasets. The results show that our algorithm not only involves a lower classification error than the conventional methods but also exhibits stable performance even under unfavorable conditions such as noise and illumination changes.

## 1. Introduction

Over the past decade, the development and dissemination of low-cost commercial visual sensors has led to numerous research related to computer vision. In order to derive a specific peculiarities from the image data acquired from such visual sensors, a diverse algorithms for pattern recognition have been developed and utilized [1,2,3,4]. The decision tree is a one of renowned predictive model that analyzes data by recursive separation according to a certain splitting criterion. Since it was originally developed to analyze data that are not readily identifiable in the fields of statistics and data mining, it has been widely used to obtain meaningful outcomes from various types of real-world data including image data [5]. Unlike other predictive models, a decision tree model offers the advantage of high generalization at a relatively low computational cost for small-scale as well as large-scale data. Moreover, it has exceptional and wide-ranging data interpretation capabilities. Numerous applications, such as recognition [1,6,7,8,9,10,11,12,13], pose estimation [14,15,16,17], edge detection [18], and biomedical tasks [19,20,21,22], have clearly demonstrated the performance benefits of tree models. Furthermore, it is easy to integrate a simple tree structure with other prediction techniques. In addition, tree models have remarkable usability and accessibility because they have already been implemented as open-source models across various platforms and environments. Consequently, they are expected to be actively used in the field of pattern recognition for addressing classification, regression, and clustering problems in the future.

**Figure 1:**
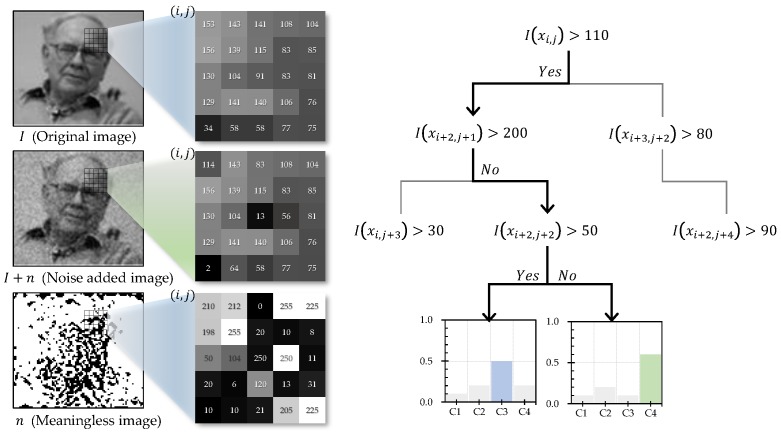
An example showing the disadvantages of conventional tree models. A trained tree that is strongly dependent on the splitting attribute is vulnerable to noise or illumination changes.

The basic tree algorithms are derived by iteratively choosing a specific attribute that best divides the data in the top-down direction. To guarantee high prediction performance, the optimal splitting attribute should be selected on the basis of divisional metrics, such as information gain and the Gini impurity. Conventional tree-based methods use a univariate system that employs only one attribute to generate each split node. However, this traditional strategy has several serious drawbacks in image classification problems. Because the selected best attribute of each split node corresponds to one pixel, the method is strongly dependent on the position and value of the corresponding splitting attribute. Thus, the tree-based approach can fall into fitting problems when modeling training data. As shown in Figure 1, another disadvantage of this approach is its sensitivity to changes in noise and illumination, even when training a large amount of data. Moreover, conventional methods that adopt lexicographical ordering in the tree induction process suffer from a critical drawback in image classification problems. Specifically, they involve semantic energy loss, which means that the content information of the image is destroyed. To overcome these limitations, various ensemble approaches [23], such as boosting, bagging, and multivariate techniques [24,25] have been introduced. Nevertheless, the fundamental problems caused by using only a few sparsely located attributes for partitioning the data have not been addressed thus far.

In this paper, we propose a new tree model, namely *L-Tree*, which learns the local area of an image and utilizes it as a single classification criterion. Unlike conventional tree-based methods, our algorithm preserves the semantic energy and classifies an image on the basis of local area similarity. Thus, it exhibits better performance than conventional tree-based methods without any image processing and is especially stable under unfavorable conditions, such as noise and illumination changes.

The remainder of this paper is organized as follows. Section 2 reviews the related work. Section 3 describes our L-Tree algorithm in detail. Section 4 presents the experimental results. Section 5 discusses the contributions of the proposed method. Finally, Section 6 concludes the paper and briefly explores directions for future work.

## 2. Background

A decision tree is a simple structural model that consists of nodes connected by edges. The growth of the tree requires training data to be learned by recursive separation from the topmost root node to the last leaf node. To select an optimal split node, all the potential attributes are repeatedly evaluated according to a specific criterion that measures their relative importance. Subsequently, each node contains the information of the best splitting attribute that is used as a criterion to identify unseen input data entering the tree. In general, the following parameters are used as a splitting criterion to search for the best attribute: information gain, gain ratio, and the Gini impurity. Numerous studies have investigated these metrics for generating tree models. Quinlan et al. [26,27] introduced the iterative dichotomiser 3 (ID3) and C4.5 algorithms, which use the concept of entropy based on information theory; two terms, information gain and gain ratio, respectively, are used to find the best attribute that minimizes the current entropy at each node. Breiman et al. [28] proposed the classification and regression tree (CART) algorithm to build a single-tree classifier using the Gini impurity; the tree is organized according to a pruning algorithm with error estimation by cross-validation. Farid et al. [29] proposed a hybrid decision tree by employing a naive Bayesian classifier. Wang et al. [30] proposed the unified Tsallis criterion decision tree (UTCDT) algorithm to generalize the splitting criteria. Tree algorithms are widely used in the field of pattern recognition because they offer several advantages over other prediction models, such as simple interpretation, high predictability at a relatively low computational cost, and easy accessibility. However, despite these advantages, trees have severe shortcomings. Their simple structure is derived by selecting only one best attribute at a time; thus, this greedy characteristic degrades performance. In particular, trees are vulnerable to fitting problems and noise.

Various studies have investigated tree models in order to overcome the above-mentioned problems. An ensemble approach that creates a strong classifier by combining multiple weak learners with the same learning algorithm has been introduced. In particular, this technique has good generalization capabilities because it does not focus on any particular instance of the training set, and it develops a model that is robust against over-fitting in the case of noisy data. For this reason, many ensemble studies have been conducted to exploit the advantages of the tree model. Breiman et al. [31] introduced a strong classifier with bootstrap aggregating (bagging) that changes the distribution of the dataset stochastically. Further, he proposed improved versions of bagging, namely random forests [32], to enhance performance through randomness and diversity by using randomly chosen feature subsets. Geurts et al. [33] proposed an extremely randomized tree that generates each split node by selecting a random cut point in the entire training data. Rodriguez et al. [34] proposed a new ensemble approach that creates new features while preserving the original properties through decomposition and reconfiguration of an instance via principal component analysis (PCA) [35]. In addition, various studies have investigated boosting [36,37,38] and multivariate splitting techniques [24,25] to improve the performance of tree models. These methods are effective in compensating for the disadvantages of classical trees and improving their generalization abilities. Nevertheless, they suffer from serious limitations in image classification problems. As base learners, most tree models use a few variates to analyze instances although they are ensembled. Hence, their performance is strongly dependent on the values and positions of the splitting attributes. Thus, the semantic information is not considered; only a small fraction of the image is used as a splitting criterion. Another problem is that the relative importance of the optimal attribute’s position decreases in the case of high-dimensional input data. Therefore, tree-based methods continue to suffer from instability as well as uncertainty due to noise and illumination changes.

In this paper, we propose a new tree induction algorithm that is generated by conserving and considering semantic information on the basis of local area learning for image classification. Toward this end, we adopt a self-organizing map (SOM) [39] algorithm and merge it with the tree model. At each node of our tree model, the splitting criterion is created by training a randomly selected sub-window image set using the SOM algorithm. The SOM, which is also known as the Kohonen network, is a type of artificial neural network (ANN) for low-level clustering analysis of high-dimensional data through self-learning. It is mainly used in applications such as data visualization [40], trend analysis [41], and segmentation [42]. Various studies have been conducted to achieve improved performance through a hierarchical strategy [43,44,45]. Our algorithm differs from these methods in the following aspects. First, our model is a type of decision tree that grows by learning the local area of images for image classification; thus, it has a different origin. Next, our algorithm can easily employ various metrics, such as information gain and the Gini impurity, which have been used in previous tree induction algorithms, because we follow the classical tree-growing scheme. Therefore, it has better data interpretation and prediction abilities than existing tree-based methods. Moreover, it can handle multiple classes. Finally, our tree model is not a deterministic model. It is derived using our own randomization techniques by adopting a random optimization method, such as random sub-window extraction and random sampled node learning. Therefore, it is possible to employ other clustering algorithms besides the SOM algorithm for our tree induction. These factors distinguish our algorithm from existing schemes. Furthermore, unlike conventional tree-based methods, our algorithm is suitable for image classification problems in terms of semantic energy conservation because the image itself is used as splitting criterion without any handcrafted feature. Although there are several attempts [46,47] which combining random sub-windows with a decision tree already have conducted to take above an advantages. However, these approaches employ a tree algorithm as a feature extractor unlike ours which utilizes a local image itself as a feature descriptor to recognize. Consequently, our proposed method does not need a further classification algorithm to recognize the image. It means our method can avoid the increased uncertainty caused by an added classification algorithm. In addition, it has good generalization capabilities based on randomization techniques. The generated L-Tree classifies data from the root node to a leaf node that stores a posterior probability by calculating the similarities of an image’s local areas. Qualitative and quantitative analyses show that the proposed algorithm exhibits better performance than previous tree-based classification algorithms and that it is effective under unfavorable conditions, such as noise and illumination changes.

## 3. Proposed Methodology

**Figure 2:**
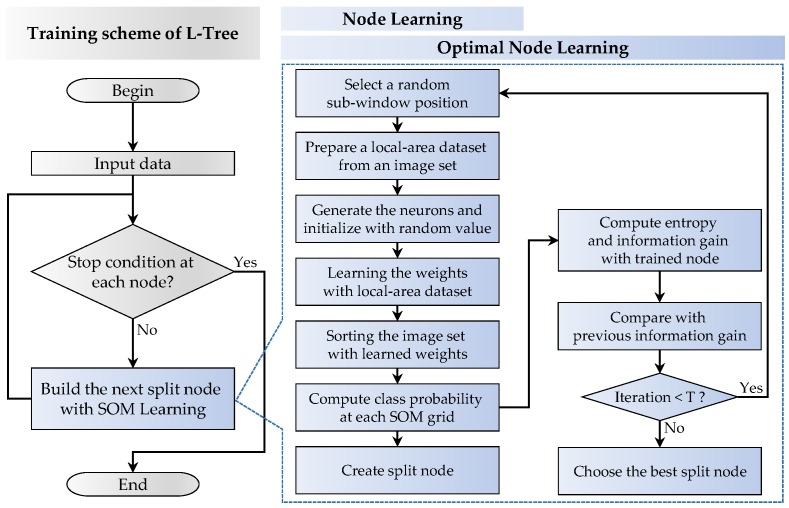
Overall flowchart of our tree induction algorithm.

In this section, we provide a detailed description of the L-Tree induction algorithm for image classification. The overall flowchart of the proposed algorithm is shown in Figure 2. The algorithm starts from the root node at the top, and it learns and creates a splitting criterion at each node in the top-down direction until it reaches the leaf node. This is known as supervised learning, which is used in conventional tree induction methods. However, it requires normalization for reducing variations of interclass data, random sub-window extraction for utilizing image features, and entropy-based optimization for improving classification performance. The details are explained in the following subsections.

### 3.1. Random Sub-Window Extraction

Let X denote image data with image width *W* and height *H* as follows:

(1) $$X=\left[\begin{array}{cccc} x_{0,0} & x_{1,0} & \cdots & x_{W-1,0}\\ x_{0,1} & x_{1,1} & \cdots & x_{W-1,1}\\ \vdots & \vdots & \ddots & \vdots \\ x_{0,H-1} & x_{1,H-1} & \cdots & x_{W-1,H-1} \end{array}\right]$$

where each $x_{ij}$ is $x\in R^{W\times H}$. Let $\bar{\bar{X}}=\left\{X_{1},X_{2},\ldots ,X_{N}\right\}$ be the training dataset containing the training objects in the form of an N×W×H matrix. Let *Y* be a vector with class labels for the data, $Y=\{y_{1},y_{2},\ldots ,y_{N}\}$, where $y_{j}$ takes a value from the set of class labels $\left\{L_{1},L_{2},\ldots ,L_{C}\right\}$. In the L-Tree, the image itself is used as a feature for image classification. Furthermore, it is used as the splitting criterion. Therefore, each node randomly extracts a set of local sub-windows x from the original image X. Here, the ratio of the size of the randomly selected sub-window to that of the original image is denoted by the scale factor s, which takes a value between 0 and 1. The extracted image x has the following form:

(2) $$x=\left[\begin{array}{cccc} x_{i,j} & x_{i+1,j} & \cdots & x_{i+W_{s}-1,j}\\ x_{i,j+1} & x_{i+1,j+1} & \cdots & x_{i+W_{s}-1,j+1}\\ \vdots & \vdots & \ddots & \vdots \\ x_{i,j+H_{s}-1} & x_{i+1,j+H_{s}-1} & \cdots & x_{i+W_{s}-1,j+H_{s}-1} \end{array}\right]$$

where $W_{s}=s\cdot W$ and $H_{s}=s\cdot H$ ($0<W_{s}\leq W$ and $0<H_{s}\leq H$). Before node learning, the selected set $\bar{\bar{x}}=\left\{x_{1},x_{2},\ldots ,x_{N}\right\}$ of local area images x has to be normalized to reduce the effects of illumination and contrast. We first convert each image $x_{k}$ in the sub-window set $\bar{\bar{x}}$ from the 8-bit format (0–255) into the float format (with a value from 0 to 1) for more accurate calculation. Subsequently, we calculate each mean value of the local area image set.

(3) $${x_{k}}^{\prime }=x_{k}-\bar{x_{k}}$$

(4) $$\bar{x_{k}}=\frac{1}{W_{s}\times H_{s}}\sum_{j=1}^{W_{s}}\sum_{j=1}^{H_{s}}x_{k,ji}$$

where $\bar{x_{k}}$ is the mean value of the local area of *k*th sub-window image of $\bar{\bar{x}}$, and it is a scalar value. Next, the original local area is subtracted by using each calculated mean value.

Through this normalization step, the sub-window image set ${\bar{\bar{x}}}^{{}^{\prime }}$ can reduce the deviation between similar classes and facilitate intensive investigation of various characteristics, such as shape and texture. Consequently, the features are robust against noise and illumination changes. The local area is used as a representation of the characteristics of the original dataset in the node learning process.

### 3.2. Node Learning

**Figure 3:**
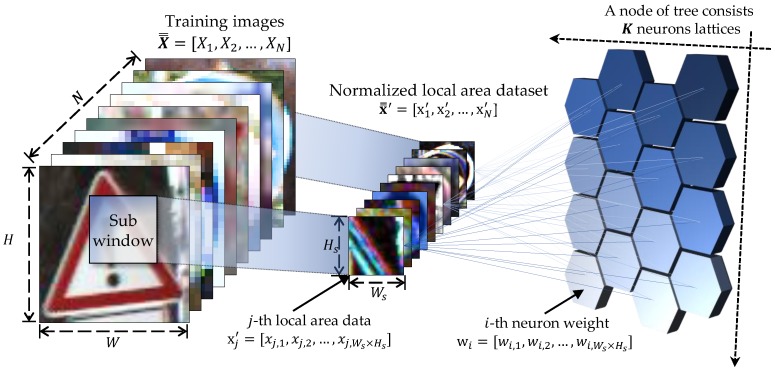
Node learning process of L-Tree.

The training dataset $\bar{\bar{X}}$ is recursively divided and learned from the topmost root node to the last leaf node using the prepared dataset ${\bar{\bar{x}}}^{{}^{\prime }}$. As shown in Figure 3, the prepared feature dataset ${\bar{\bar{x}}}^{{}^{\prime }}$ is autonomously learned using a self-organizing map (SOM) algorithm, which yields the split nodes. The conventional SOM algorithm usually consists of $K_{r}\times K_{c}$ neurons located in a two-dimensional cell grid. The *i*th neuron has a *D*-dimensional weight vector $w_{i}=\left(w_{i1},w_{i2},\ldots ,w_{iD}\right)$, where i=1,2,…,K $\left(K_{r}\times K_{c}=K\right)$. The initial values of all the weight vectors W are randomly given over the input space. The range of the elements of the *D*-dimensional input data $x_{j}=\left(x_{j,1},x_{j,2},\ldots ,x_{j,D}\right)\left(j=1,2,\ldots ,N\right)$ is assumed to be from 0 to 1. When a training local area image $x_{j}$ is fed to the network, the winner $\hat{i}$ is the neuron whose weight vector is closest to the training vector $x_{j}$, which can be denoted as

(5) $$\hat{i}=\underset{i}{\arg\max}\left|\left|w_{i}-x_{j}\right|\right|\;$$

where $\left|\left|\cdot \right|\right|$ is the Euclidean distance. Then, the weight vector of the winner and its neighbors can be updated as

(6) $$w_{i}\left(t+1\right)=w_{i}\left(t\right)+h_{c,i}\left(x_{j}-w_{i}\left(t\right)\right)$$

(7) $$h_{c,i}=\alpha \left(t\right)\exp\left(-\frac{{\left|\left|r_{c}-r_{i}\right|\right|}^{2}}{2\alpha {\left(t\right)}^{2}}\right)$$

(8) $$\alpha \left(t\right)=\alpha \left(t\right)\exp\left(-\frac{1}{\tau }\right)$$

where *t* is the learning step and $h_{c,i}\left(t\right)$ is the neighborhood function, which is defined by our algorithm as shown above. Further, $\left|\left|r_{i}-r_{c}\right|\right|$ is the distance between the map nodes *c* and *i* on the map grid, and α(t) is the learning rate. The convergence speed of the SOM depends on the values of the convergence variables α and τ. Basically, α represents the degree of influence on other cells, and τ determines the rate of convergence of the learning rate. Here, α(t) is reduced to Equation (8) as the iteration proceeds to guarantee convergence of learning.

**Figure 4:**
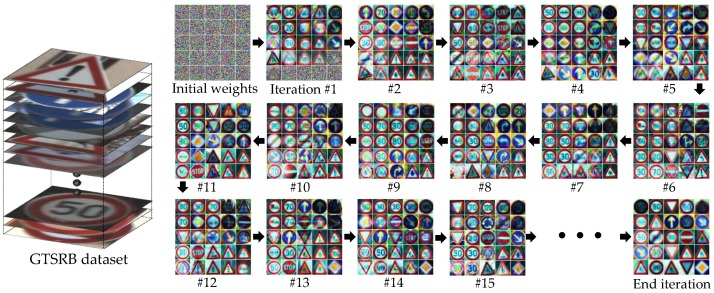
Visualization of weights at root node using GTSRB dataset. The initial weights of each neuron learned with iteration processing.

When each iteration is performed for weight learning, only the data obtained by randomly extracting the square root from the total number of learning data *N* of the node is learned. This is done to not only avoid the local minima problem in the learning process but also increase the computational efficiency. As the iteration progresses, the weight is learned to contain the information of the local-area dataset. The value to be stored in each weight differs according to the class distribution of the dataset to be trained. Figure 4 shows a visualization of the SOM weights in the training process when the scale factor s is 1.0 and the cell size is 5×5 at the root node. It illustrates how the SOM is learned through iteration. Next, we classify the training dataset into neurons with the closest values by weighting similarities with the trained weight values using the Euclidean distance. The distribution of the training dataset can be used to calculate the class probability of each neuron. As a result, the *i*th neuron stores the trained weight value $w_{i}$ and the probability from the histogram. Let $L_{C}$ be a class label, and let $P(L_{C})$ be the probability of the class, which can be obtained by calculating the relative frequency from the histogram. The node learning process proceeds and the tree continues to grow until the termination condition is satisfied. The termination condition is one of the following:

1. the number of training data to train a single node is lower than a certain threshold,
2. the depth of the tree exceeds a certain threshold,
3. the entropy of the set of classified training samples is 0 or 1.

Thus, the proposed algorithm learns the local image with randomization characteristics, thereby decreasing the position and value dependency on a few pixels and improving the generalization ability. The generated prediction model starts from the root node for a given test sample, measures similarity with all the neurons’ weights at each node, and branches to the next node that has a similar property. This process is repeated cyclically until the leaf node is reached, and the last neuron returns the stored probabilities. The Algorithm 1 shows the pseudo-code of the proposed algorithm.
**Algorithm 1** Training algorithm for L-Tree. **Input**
$\bar{\bar{X}}$: the training image set which consist of $\left\{X_{1},X_{2},\ldots ,X_{N}\right\}$ (an N×W×H matrix)Y: the labels of training image set (an N×1 matrix)*s*: the ratio between local area and original image ($W_{s}=s\cdot W$, $H_{s}=s\cdot H$)*K*: the number of neurons in one SOM ($K=K_{r}\times K_{c}$)$\left\{L_{1},L_{2},\ldots ,L_{C}\right\}$: the set of class labels**Output**: a single L-Tree
**Build a L-Tree**
$\left(\bar{\bar{X}}\right)$
1:Extract random local-area position $\left(x_{st},y_{st}\right)$2:Normalize by subtracting the mean, ${\bar{\bar{x}}}^{{}^{\prime }}$3:**Node learning** (${\bar{\bar{x}}}^{{}^{\prime }}$)4:Split $\bar{\bar{X}}$ into subset ${\bar{\bar{X}}}_{1},{\bar{\bar{X}}}_{2},\ldots ,{\bar{\bar{X}}}_{K}$ according to the similarity with *K* trained neurons.5:Calculate class probability from relative frequency of each samples and save it to the neurons.6:For j=1,2,…,K inspect a terminalcriteria7:If 6 satisfying stop the node learning, else then **Build a L-Tree** (${\bar{\bar{X}}}_{j}$).**Node learning**
$\left({\bar{\bar{x}}}^{\prime }\right)$
1:Generate new *K* neurons weight W(t=0) and initialize with random values before learning2:**do**3:  Select randomly $n(=\sqrt{N})$ samples from ${\bar{\bar{x}}}^{{}^{\prime }}$4:  $w_{i}\left(t+1\right)=w_{i}\left(t\right)+h_{c,i}\left(x_{j}-w_{i}\left(t\right)\right)$          ▹ Update a neuron’s weights5:  $\alpha \left(t\right)=\alpha \left(t\right)\exp\left(-\frac{1}{\tau }\right)$                   ▹ Update a learning rate6:  t=t+17:**while** until W(t)≠W(t−1)

### 3.3. Optimization

In this subsection, we discuss how to select an optimal node. The existing tree induction algorithm was intended to create only one or several optimal partition attributes that can effectively divide the dataset in a single instance. In pixel-based classification, random classification criteria might generate meaningless nodes. The proposed algorithm regards the local area as a property for division. In contrast to the conventional method, our method ensures a certain performance level by using totally random extraction, because the image’s semantic information is sufficiently preserved. However, it is obvious that unidirectional randomization has performance limitations. Hence, it is necessary to find an optimal node to divide the data effectively. We use indexes based on information theory, entropy, and information gain for node optimization. Equation (9) defines our objective function for finding the optimal node.

(9) $$\hat{W}=\underset{W}{\arg\max}\;IG\left(\bar{\bar{X}}\right)$$

where W represents the weight of the SOM that best classifies the training samples, and IG is a number indicating how well the given sample can be categorized as the information gain. As a result, the neurons of one node may contain samples belonging to several classifications. In this manner, the SOM repeats the learning of the nodes and keeps the samples of the same class in the child node as far as possible. To solve this optimization problem, we use random optimization. This is a method of selecting the optimal SOM weights that exhibit better splitting performance while repeatedly selecting the position of the sub-window with a uniform probability. Consequently, it is necessary to calculate the entropy that can measure the distribution of the data contained in one node. Entropy is an index of the purity of a dataset; it calculates the uncertainty contained in a probability distribution. The entropy can be expressed in a generalized form in statistical mechanics as follows.

(10) $$H\left(\bar{\bar{X}}\right)=-\sum_{i=1}^{n}P\left(X_{i}\right)\log_{2}P\left(X_{i}\right)$$

where *P* is the probability of each class that is obtained from the histogram. Further, *H* is 0 when the distribution of data by a class of $P(x_{i})$ is small, and it approaches 1 when in the opposite case. The information gain can be obtained by this entropy difference between the parent node and the child nodes, as expressed in Equation (11). The information gain denotes the reduction in the entropy when the sample is separated on the basis of a specific property. It shows how effectively the learned SOM’s weights can distinguish the data. In addition, IG can be used to evaluate the relative importance of the weight of the SOM created by learning the sub-window selected with uniform probability. A large value of the information gain indicates that the learned weights can better classify the data.

(11) $$IG\left(\bar{\bar{X}},W\right)=H\left(\bar{\bar{X}}\right)-\sum_{v\in w}\frac{\left|\{X\in \bar{\bar{X}}|x^{{}^{\prime }}=v\}\right|}{|\bar{\bar{X}}|}\cdot H\left(\left\{X\in \bar{\bar{X}}|x^{{}^{\prime }}=v\right\}\right)$$

**Figure 5:**
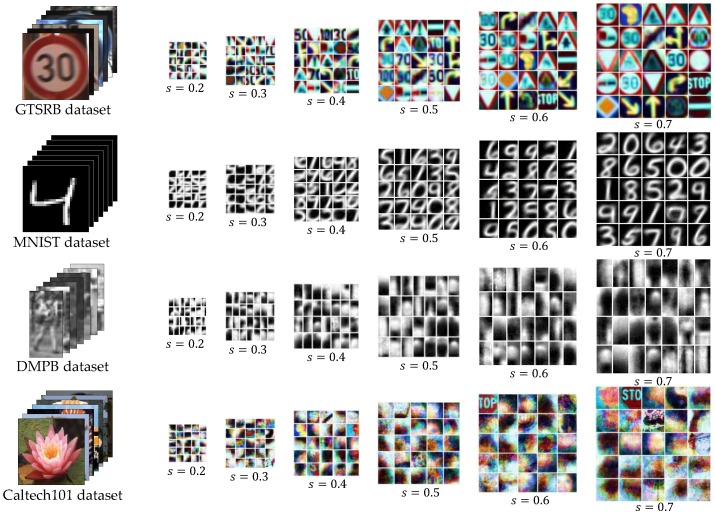
Visualization of SOM weights at root node with different scale values.

The optimization proceeds by the specified iteration *T*, and the classification performance is enhanced. Moreover, the size of the tree becomes compact owing to the suppressed generation of meaningless nodes. The Algorithm 2 and the Figure 5 show the pseudo-code of the tree induction algorithm generated by the optimization technique and the visualization results with variations of scale.
**Algorithm 2** Training algorithm for an Optimized L-Tree. **Prepare**
$W_{best}$: the best SOM weights to classification the object$IG_{best}$: the information gain when the SOM weights has best classification ability**Build an optimized L-Tree** ($\bar{\bar{X}}$) 1:for t=1:T2:  Extract random local-area position $\left(x_{st},y_{st}\right)$3:  normalize by subtracting the mean, ${\bar{\bar{x}}}^{{}^{\prime }}$4:  Learning SOM weights $W_{t}$ through the **Node learning** ($\bar{\bar{x}}$)5:  Split $\bar{\bar{X}}$ into subset ${\bar{\bar{X}}}_{1},{\bar{\bar{X}}}_{2},\ldots ,{\bar{\bar{X}}}_{K}$ according to the similarity with *K* trained nuerons.6:  Calculate parent and child entropies $H(\bar{\bar{X}})$ when the dataset is classified with W.7:  Calculate Information gain ($IG_{t}\left(\bar{\bar{X}},W_{t}\right)$) from the entropy.8:  **if** $IG_{t}>IG_{best}$9:    $W_{best}=W_{t}$10:    $IG_{best}=IG_{t}$11:  **End if**12:Endfor13:for j=1,2,…,K check a terminalcriteria14:If 6 satisfying stop the node learning, else then **Build an optimized L-Tree** (${\bar{\bar{X}}}_{j}$).

### 3.4. Bagging Approach

**Figure 6:**
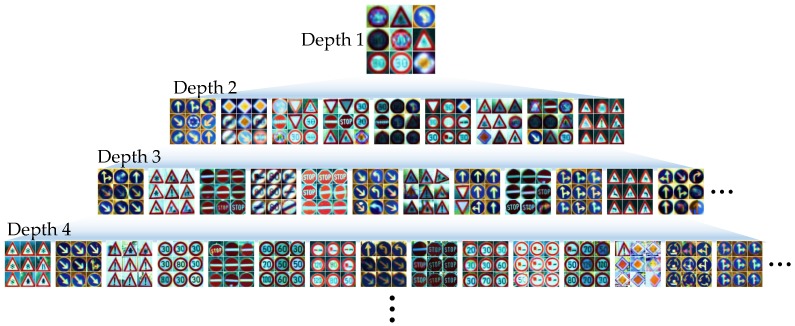
Visualization of node in one Opt-L-Tree with different depths of GTSRB datasets.

The proposed algorithm is a type of tree that uses the local area information of an image for classification. It can be easily integrated with ensemble techniques such as the conventional tree model. Conventional ensemble techniques create a strong classifier by training several weak learners through the redistribution of training datasets. In this study, we attempt to improve the generalization ability by using bagging approaches and employing the proposed tree model as a base learner. Given a training set $\bar{\bar{X}}$ of size *N*, bagging generates *L* new training sets, each of size $n(=\sqrt{N})$, by uniform and redundant sampling. Let $D_{k}$ denote a weak classifier for each training set; all classifiers are trained in parallel. Finally, the strong classifier D, which consists of $D_{1},D_{2},\ldots ,D_{L}$, makes the final decision through a majority vote. Bagging has the advantage of decreasing the predictive correlation of each tree and improving the generalization performance. Figure 6 shows a visualization of the weights stored in each node of the L-Tree created using the Germany traffic sign dataset (GTSRB). For simplicity, we set the scale factor s to 1 and the SOM size to 3×3. As the tree becomes deeper, images can be gradually classified into the same class. Visualization of node in one Opt-L-Tree for other datasets, see Appendix B.

## 4. Experiments

This section presents the results of experiments conducted to evaluate our algorithm. To validate the performance from various aspects, quantitative evaluation was carried out under different conditions and settings.

### 4.1. Dataset

**Figure 7:**
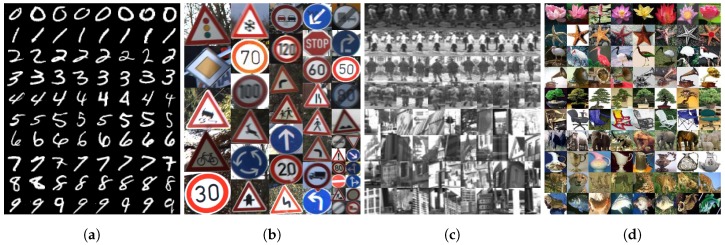
Four different datasets for evaluation: (**a**) MNIST dataset; (**b**) GTSRB dataset; (**c**) DMPB dataset; and (**d**) Caltech101 dataset.

Four well-known datasets were used to evaluate the performance of the proposed algorithm as shown in Figure 7. Each of these datasets was selected in consideration of the classification problem, by taking various conditions of multiclass tasks into account, such as digits, traffic signs, and general objects. The detailed specifications of the datasets used in the experiments are as follows. The Modified National Institute of Standards and Technology (MNIST) dataset [48] consists of 60k training images and 10k test images. It includes 28 × 28 images per image, divided into 10 classes from 0 to 9. The German Traffic Sign Recognition Benchmark (GTSRB) dataset [49] consists of traffic sign images. It includes around 39k images and 13k test images. Further, it consists of 43 classes. Each class has a different number of images, varying illumination and pose, and is composed of images of various resolutions, ranging from 28 × 28 to 40 × 40. We also used the the Daimler Mono Pedestrian Benchmark (DMPB) dataset [50] consists of around 29k training pedestrian images and 14k test negative images of size 18 × 36 for mono classification tasks. Finally, the Caltech101 dataset [51] was chosen to evaluate our proposed method. It consists of about 9k images in 101 classes including vehicles, face, airplane, etc. Each class includes 30 to 800 images of different sizes, and each sample has significant a variety of shape and texture. For validation, the image of MNIST and DMPB were used without any changed. However, the GTSRB and Caltech101 dataset were resized to 36 × 36 color images regardless of the image quality. In case of employing the Caltech101 dataset, we randomly shuffled and partitioned the whole dataset into 30 training samples per class and no more than 50 images to test per class.

### 4.2. Evaluation Method

We conducted experiments for quantitative validation by comparison with other classification algorithms. For comparison, we implemented nine different tree-based methods: C4.5 [27], trees constructed by information gain (UTCDT1) and gain ratio (UTCDT2) based on the unified Tsallis entropy [30], hybrid tree using a naive Bayesian classifier [29] (NBT), CART [28], bagging tree (Bag(cart)) [31], random forest (RandF) [32], rotation forest (RotF) [34], and extremely randomized tree (ERT) [33]. We examined the ensemble performance with each single-tree method. Further, we constructed additional ensemble methods with single-tree algorithms, namely Bag(c4.5), Bag(utcdt1), Bag(utcdt2), and Bag(nbt). To evaluate the performance under ranging environmental conditions, all the tree algorithms had a maximum tree depth of 10. Further, the minimum number of samples at the last node was set to 10 and pruning was not performed. The ensemble number was set to 100 for all multi-tree cases included our methods. For UTCDT1 and UTCDT2, the tuning parameter *q* value was set to 1.1. RotF and RandF were constructed using the CART method. In the case of RotF, the number of features in a subset, *M* was set to 10, and the feature was reconstructed through PCA with retained features. The reconstructed features were trained as a decision forest CART. For the RandF, the size of the randomly selected subset of features at each node was set to the square root of the total number of features. We used a single L-Tree and a single Opt-L-Tree as our proposed algorithm for comparison, and we also used the bagging technique Bag(L-Tree) and Bag(Opt-L-Tree) for evaluation. Both of single and ensemble cases applied α = 0.5, τ = 500, and cell size (K) = 3 × 3, and optimized iteration = 20 while scale factor(s) was set to 0.8 for single L-Tree and scale to 0.3 for Bag(L-Tree) respectively.

**Figure 8:**
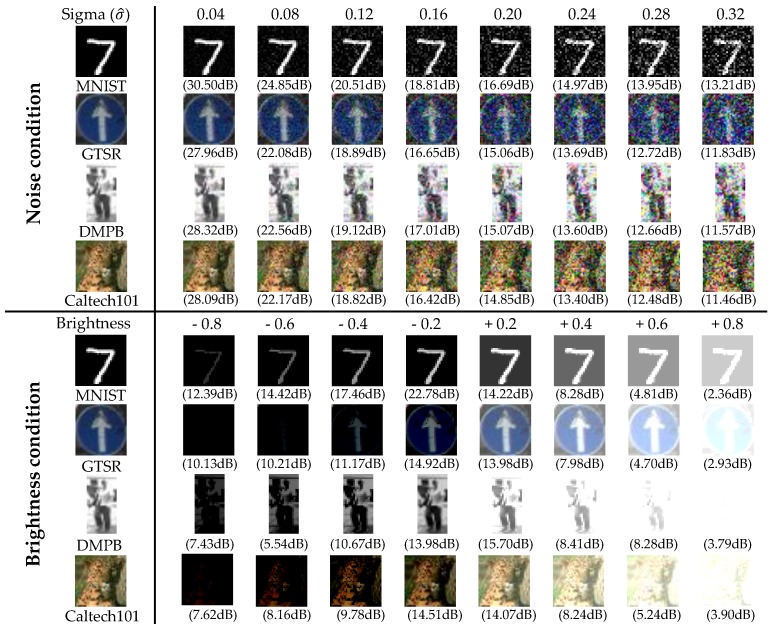
Images under different conditions with variations of Gaussian sigma and brightness.

The experiments were conducted under normal conditions as well as under unfavorable conditions such as noise and illumination changes. To generate the unfavorable conditions, we used Gaussian noise with different sigma values and added a control variable for noise to the image. The image brightness was changed by adding or subtracting a specific scalar value to or from the entire image. As the sigma value of the Gaussian noise increases with the image brightness, the image quality deteriorates. To measure the quality of the noisy and brighten the image, we used the peak signal-to-noise ratio (PSNR). Figure 8 shows some test examples of the four different datasets with changes in the sigma value and brightness. All the results reflect the average value after five repetitions.

To investigate the effect of the parameters used in the proposed algorithm on the classification performance, we used the MNIST dataset. There are six parameters to be evaluated: scale factor s that determines the sub-window size, neuron number K, tree structural parameters (tree depth and number of samples at leaf node), and learning parameters of the SOM algorithm (α and τ). The qualitative evaluation was performed using the classification error rate (CER), which varies according to the parameters in the case of the single L-Tree and Opt-L-Tree. We also performed ten-fold cross-validation [52] to avoid fitting problems. The cross-validation technique was applied to the training set, and we carried out training and testing with the selected parameters. In each graph, the cross-validation error (CV-error) and standard error mean (SEM) are indicated, and the training error (TR-error) and test error (TE-error) are also represented. For accurate investigation, when changing one parameter, the remaining parameters were fixed.

Next, we examined the optimization process. To investigate the effectiveness of our approach, we proposed two metrics: error decrement and node savings.

(12) ithErrorDecrement(EDi)=CERiCER0

(13) ithNodeSavings(NSi)=NNiNN0

where $CER_{i}$ and $NN_{i}$ represent the classification error rate and the number of nodes in one L-Tree at the *i*th iteration, respectively. The ED indicates the performance improvement through optimization, which decreases gradually as the iteration progresses. Further, NS represents the spatial efficiency as the number of nodes saved through the optimization process, which increases as the iteration progresses. Each L-tree and Opt-L-Tree train all 60k data of MNIST with variations of the cell size and sub-window ratio.

In order to explore the actual implementation time of the L-Tree compared with other algorithms, we employed MNIST dataset and investigated the time required to construct a sinle tree by calculate the average time of an one hundreds produced trees. In addition, we measured the classification time spent per an image using the same dataset. The default parameters for the parameter, optimization, and computational complexity experiments are as follows: scale factor(s) = 0.3, cell size (K) = 4 × 4, maximum tree depth = 10, minimum number of samples in leaf node = 10, α = 0.5, τ = 500, and optimizated iteration = 20. We also analyzed the effectiveness of the several parameters under the ensemble condition on GTSRB dataset. To analyze the effect on the number of trees, we excluded the depth of the tree from the termination condition, set the number of samples of the last node to 2, and recorded CAR by increasing the number of trees from 5 to 1000.

Because our algorithm is not deterministic, the results are different in every instance. Therefore, all the values shown in the graph are calculated by averaging the number of nodes and the error rates obtained by repeating the experiment five times. All the experiments have been implemented in C++ language utilizing the OpenCV [53] library. evaluated on a desktop computer running Windows 10 with Intel(R) Core(TM) i7-3770 (3.2 GHz) and 16 GB memory.

### 4.3. Experimental Result

#### 4.3.1. Normal Environmental Condition

As shown in Table 1 and Table 2, our Opt-L-tree exhibits the best performance on all the datasets. In the case of the MNIST dataset, nearly all the methods show good performance. However, the results on the other three datasets vary significantly since the GTSRB, DMPB, and Caltech101 datasets contain texture information as a major element in addition to shape. However the CAR of Bag(Opt-L-Tree) was the highest in all the datasets. A comparison of the single-tree methods showed that C4.5 exhibited the worst performance. Both single and multiple tree cases, the CAR in the L-Tree is presented much higher than the other method. Interestingly, an unoptimized L-tree shows better CAR than other single-tree methods and ensemble methods. It implies that the split criterion limits the classification performance when using less information. Among the ensemble models, the simple bagging tree algorithms, such as Bag(c4.5), Bag(utcdt1), Bag(utcdt2), and Bag(nbt), showed poor performance. The other ensemble algorithms, namely RandF, ERT, and RotF, showed good performance, but they were not able to outperform the L-tree. It represents the necessity for the further step to improve generalization ability in addition to the way diversity is imposed through datasets.

#### 4.3.2. Noisy Condition

**Figure 9:**
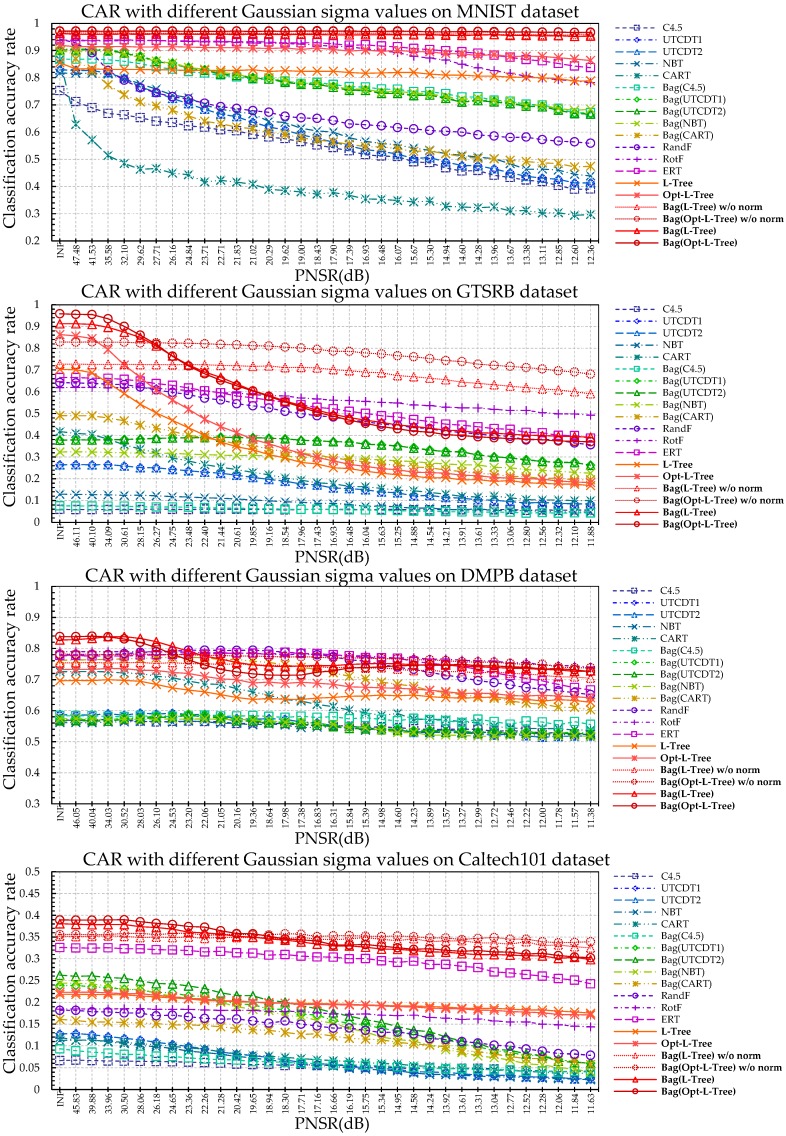
CAR Comparison with other algorithms in noisy conditions for four different datasets.

Through CAR comparison with other algorithms under noisy conditions for the four different datasets, the conventional tree methods exhibited severe performance degradation even at a PSNR of 30–40 dB, which is difficult to identify with the naked eye. In the case of GTSRB and DMPB dataset, ERT and RotF showed good performance in some sections. However, our algorithm showed better performance in most intervals as shown in Figure 9. The proposed L-Tree-based methods, ERT, and RotF have a relatively robust to the noise than other methods due to its non-dependent properties of only a few number of split criterions. However, despite the loss of information for an image with added noise, the L-Tree-based method generally exhibits a good classification rate. In particular, the MNIST dataset shows no significant performance degradation until the PSNR is approximately 11 dB.

#### 4.3.3. Varying Brightness Condition

**Figure 10:**
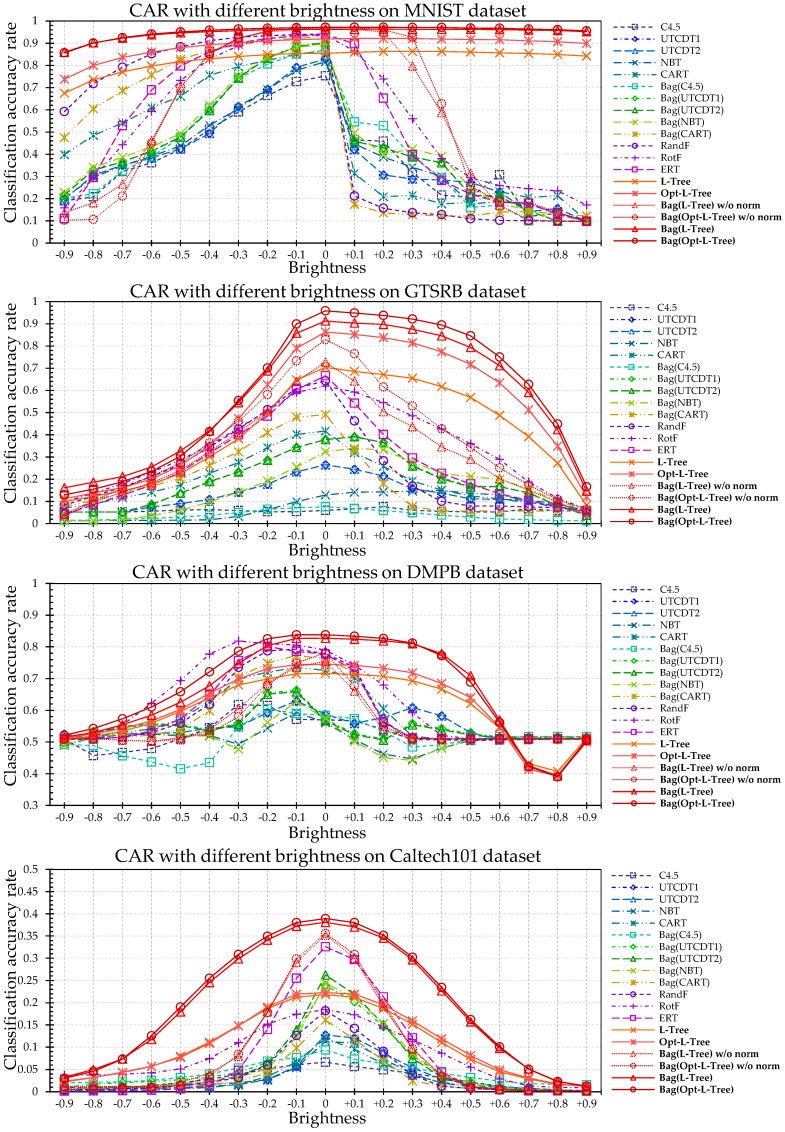
CAR comparison with other algorithms under illumination changes for four different datasets.

As shown in Figure 10, the proposed L-Tree-based methods showed excellent performance in nearly the entire area on all the datasets. Thus, RandF, RotF, and ERT are somewhat robust using ensemble techniques, but the performance degradation is noticeable in the case of extreme image information loss. The basic single L-Tree also shows partly robust results with respect to changes in illumination compared to existing tree-based ensemble models. In particular, our algorithm does not show significant degradation in the case of the MNIST dataset, and a single L-Tree shows better performance than some of the ensemble methods in extremely difficult cases. Because hierarchically chosen local-area images at the test phase are adjusted to the learned neurons data through the normalization process. Further, C4.5, which shows the worst classification rate, and existing tree-based models including it, are highly sensitive to illumination changes.

#### 4.3.4. Parameter Influences on the Single L-Tree

**Figure 11:**
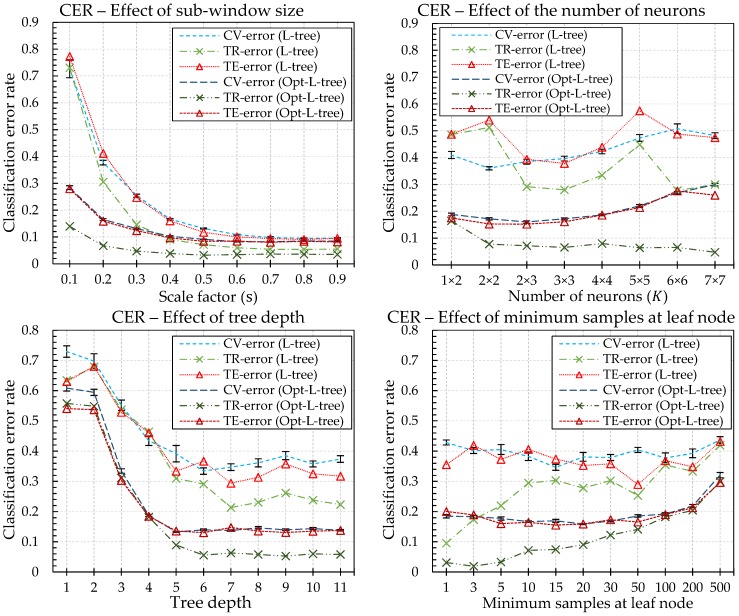

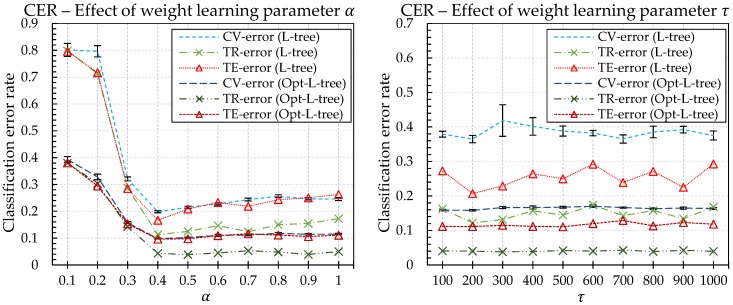
Classification results of single L-Tree and Opt-L-Tree under different parameter changes. All values except τ have a significant effect on the result, and they all determine the complexity of the model of the generated tree.

The results in Figure 11 show that all six parameters affect the results. The tree depth and the minimum number of samples of the last node, which determine the structure of the tree, have similar patterns compared with the conventional tree model. As the depth increases and as the minimum number at the leaf node decreases, the performance improves. However, overfitting of the training datasets occurs and the classification rate becomes poor above a certain threshold. The sub-window ratio and cell size, which are necessary for tree node training, also affect the results as much as the parameters of the tree structure. The L-Tree has the best CER when both parameters have proper values. The training parameters of the SOM, namely alpha and tau, do not have a significant effect on the other parameters. However, in the case of alpha, the weight learning of the SOM affects the neighboring neurons. If the value is too small, the neurons of the SOM will not be properly learned and the performance will be poor. In the case of a tree that has undergone an optimization process, the results obtained by adjusting the parameters that determine the cell size and the sub-window size are different from those of the L-Tree. However, they also have a similar tendency compared to the remaining parameters.

#### 4.3.5. Optimization

**Figure 12:**
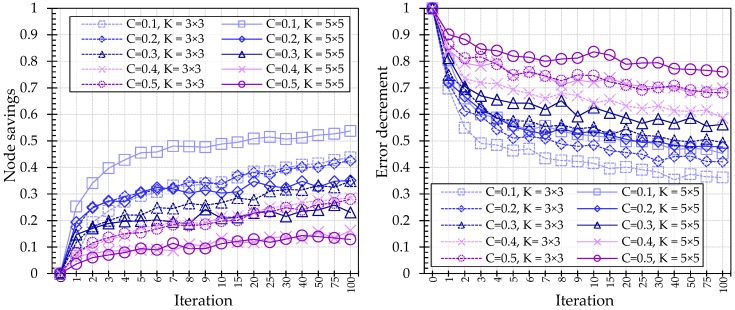
NS and ED results of single L-Tree with variations of optimized iteration.

The effect of optimization can be seen in all the cases. As shown in Figure 12, the NS and ED represent the relative ratios to non-optimized trees. In particular, a model with a small local area shows the effect of optimization more clearly. Conversely, the effect of optimization is relatively small in the case of large trees with sub-windows. This tendency is also exhibited by the cell size. As the iteration progresses, all the cases become more compact and show better classification performance. This result demonstrates that the performance of the L-Tree can be improved through optimization.

#### 4.3.6. Computational Complexity

The computational complexity at test time for a single L-tree of scale size s, feature dimension *L*, number of neurons *K*, and maximum depth *D* is O(sLKD) in comparison with the conventional tree method is O(D). The training cost to generate a single L-tree can be expressed by O(sLKDTN) where an iteration number is *T* and a training sample number is *N*. Nevertheless, the substantive computational time in the training and testing phases much lower can be lower if the tree depth is not reached to the maximum depth or a produced tree is not balanced. As shown in Table 3, the L-Tree algorithm demands more longer time to classify than other traditional tree methods. However, the lower classification ability at the parent node implies that the tree need to be learned more. Therefore, There was not a significant deviation from other methods in the training phase. The RotF was slowest in the testing phase due to the computation process of PCA.

#### 4.3.7. Parameter Influences on the Ensemble Condition

**Figure 13:**
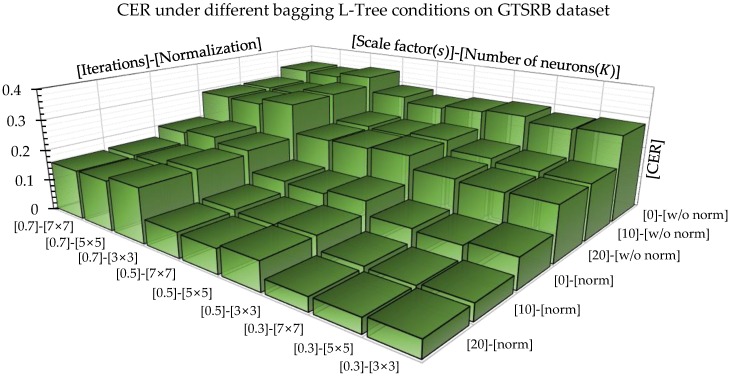
The CER comparison under various bagging L-Tree condition with changing the scale factor, iteration number, number of neurons, and normalization.

The results in Figure 13 show a study on affecting factors of the 100 ensemble L-Trees. To enhance the effectiveness of an ensemble, it is important to build different trees while retaining the same classification tendency. The CER with different conditions show that there are many factors that affect classification capability beyond the bootstrap aggregation method. First of all, the performance improves as the number of optimization iteration increases. This significantly differs depending on the number of times and have a similar performance from a higher than a certain number of times. In contrast to the single tree case, the classification accuracy represented a tendency that becoming higher when the ensemble model has a smaller scale factor. The sub-window size has a decisive effect on the classification performance than other factors in particular. Because the smaller scale factor ensures lower correlation between the trees. Interestingly, the normalization process further had a significant impact on performance. It can be interpreted that the local-area normalization allows focussing on learning only meaningful information from images obtained from various conditions.

## 5. Discussion

Despite their various advantages, decision trees are vulnerable to fitting problems and noise in image classification tasks, because they have a simple structure based on the selection of only one optimal attribute at each split node. An ensemble model has been proposed as a solution to these issues, but its performance is limited under unfavorable conditions. The tree as a basic trainer does not take the semantic information of the image into account, and it uses using only a fraction of the image information. In addition, the relative importance of the positions of optimal partition attributes decreases in the case of high-dimensional feature vectors of the training data. Thus, existing algorithms show limited classification performance owing to their uncertainty, especially under unfavorable conditions such as noise and illumination changes. The experimental results verified that the image classification performance of the proposed random local area learning method is superior to that of conventional tree-based methods.

In contrast to the conventional tree induction algorithm, which uses only a few scalar values from a small part of an image, our method uses the local image area as the splitting criterion. The local area (i.e., sub-window), which is the basis of partitioning, is extracted randomly, and it is used as a feature for training an L-Tree. The proposed method reduces the dependence of the location of the best attribute of the existing pixel-based tree classifier, thus maintaining a certain level of performance in terms of noise and illumination variations. Compared with various methods developed to overcome the disadvantages of the existing single decision tree, our method preserves the energy of the entire image through an ensemble technique. This approach is advantageous in terms of utilization of the semantic energy. Moreover, this method can perform better than conventional tree methods in normal conditions. The experimental results demonstrated that our method overcomes the limitation of strong dependence on the value and location of scattered split attributes. Consequently, the results showed good performance under unfavorable conditions such as noise and illumination changes.

Furthermore, the optimization step enhances the classification performance by providing directionality in the sub-window selection process. In particular, when the sub-window size of an image is small, the efficiency increases because small windows have a smaller amount of information than large windows. In the case of a tree using a small-sized window, unplanned randomization means that there is a high probability of creating meaningless nodes. This is because, as the amount of information used as a sorting feature decreases, the importance of existing optimal sub-window locations increases. The ensemble model is fully randomized to exhibit the best performance when different trees are grown, because the size of the local window used is reduced, which decrease the correlation coefficient between the generated trees; this is more efficient in terms of diversity. Thus, our Opt-L-Tree with a small sub-window could be the best choice when it becomes a weak classifier for bagging. On the other hand, it is observed that the depth of the existing tree model and the minimum number of samples of the leaf node, especially the sub-window ratio and cell size, affect the performance of the L-Tree. Moreover, because the effect of the parameters depends on the presence or absence of optimization, it is necessary to select the parameters by considering the complexity of the model. Further theoretical research is required in this regard.

## 6. Conclusions

We proposed a new tree model and learning framework that learns information from local image areas for classification. The proposed method was shown to satisfactorily overcome the drawbacks of existing methods. Moreover, it has scope for further performance improvement. In other words, the proposed method overcomes the disadvantages of insufficient information of the partition property and improves the performance. Furthermore, we evaluate our method on traffic sign dataset and pedestrian dataset and it shows superior performance even under unfavorable conditions, such as noise and illumination changes. It means our method could be applied to real-world applications which use the visual sensor, such as autonomous driving assistance system (ADAS) and object detection. To derive the proposed tree model, we randomly selected sub-windows and trained the information by using the SOM algorithm. We also used random sampled optimization for optimal node learning. The L-Tree thus generated can hierarchically classify image data on the basis of similarity in terms of the weight of each node’s neurons. Finally, we tried to improve the performance by using a bagging technique. Our experimental results showed that the image classification performance is stable under various conditions. Unlike conventional methods, our method is suitable for image classification in terms of semantic energy conservation because the image itself is used as a separation criterion.

In the future, we plan to explore the advantages of L-Trees on more complex datasets and under more complex conditions by using a variety of pre-processing or optimization techniques. In addition, we plan to improve the efficiency of node learning through various clustering techniques. Finally, our method can be easily applied to other ensemble techniques. Therefore, we also plan to study techniques such as boosting.

## Figures and Tables

**Table 1:** CAR of different single-tree methods on four datasets.

Datasets	C4.5	UTCDT1	UTCDT2	NBT	CART	L-Tree	Opt-L-Tree
MNIST	0.7893	0.8249	0.8286	0.8149	0.8648	0.8646 ± 0.0092	**0.9156** **± 0.0027**
GTSRB	0.0592	0.2644	0.2644	0.1290	0.4169	0.7144 ± 0.0057	**0.8463** **± 0.0040**
DMPB	0.5701	0.5872	0.5872	0.5613	0.7258	0.7056 ± 0.0079	**0.7421** **± 0.0043**
Caltech101	0.0667 ± 0.0066	0.1316 ± 0.0040	0.1271 ± 0.0040	0.1127 ± 0.0132	0.1190 ± 0.0230	0.2182 ± 0.0103	**0.2228** **± 0.0136**

**Table 2:** CAR of different ensemble methods on four datasets.

Datasets	Bag (C4.5)	Bag (UTCDT1)	Bag (NBT)	Bag (CART)	RandF	RotF	ERT	Bag (L-Tree)	Bag (Opt-L-Tree)
MNIST	0.8684 ± 0.0026	0.9053 ± 0.0073	0.9026 ± 0.0049	0.9322 ± 0.0042	0.9417 ± 0.0067	0.9366 ± 0.0021	0.9389 ± 0.0000	0.9630 ± 0.0066	**0.9717** **± 0.0025**
GTSRB	0.0760 ± 0.0278	0.3779 ± 0.0051	0.3234 ± 0.0043	0.4910 ± 0.0025	0.6428 ± 0.0058	0.6196 ± 0.0049	0.6658 ± 0.0000	0.9127 ± 0.0041	**0.9583** **± 0.0052**
DMPB	0.5839 ± 0.0079	0.5711 ± 0.0081	0.5744 ± 0.0043	0.7624 ± 0.0033	0.7790 ± 0.0039	0.7901 ± 0.0017	0.7767 ± 0.0000	0.8272 ± 0.0038	**0.8384** **± 0.0039**
Caltech101	0.0933 ± 0.0165	0.2386 ± 0.0680	0.2438 ± 0.0185	0.1609 ± 0.0306	0.1816 ± 0.0461	0.1843 ± 0.0335	0.3260 ± 0.0072	0.3806 ± 0.0176	**0.3896** **± 0.0146**

**Table 3:** Computational time of different tree methods for train/test phase.

Phase	C4.5	UTCDT1	NBT	CART	RandF	RotF	ERT	L-Tree	Opt-L-Tree
Train (s)	28.62	14.58	19.33	10.68	0.49	37.51	3.14	10.32	119.98
Test (µs)	3.14	2.28	2.12	1.96	1.42	417.37	0.74	17.02	13.56

**Figure A1:**
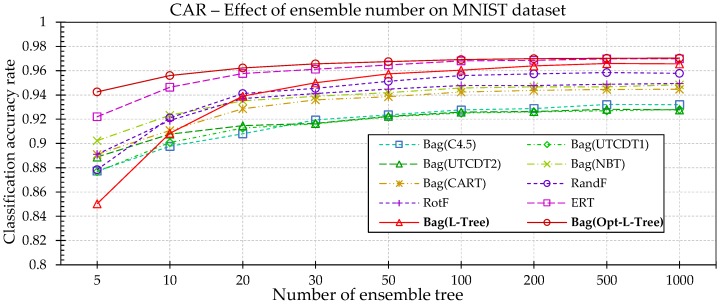
The CAR comparison with other ensemble algorithms by increasing the fully-developed tree number on MNIST dataset.

**Figure A2:**
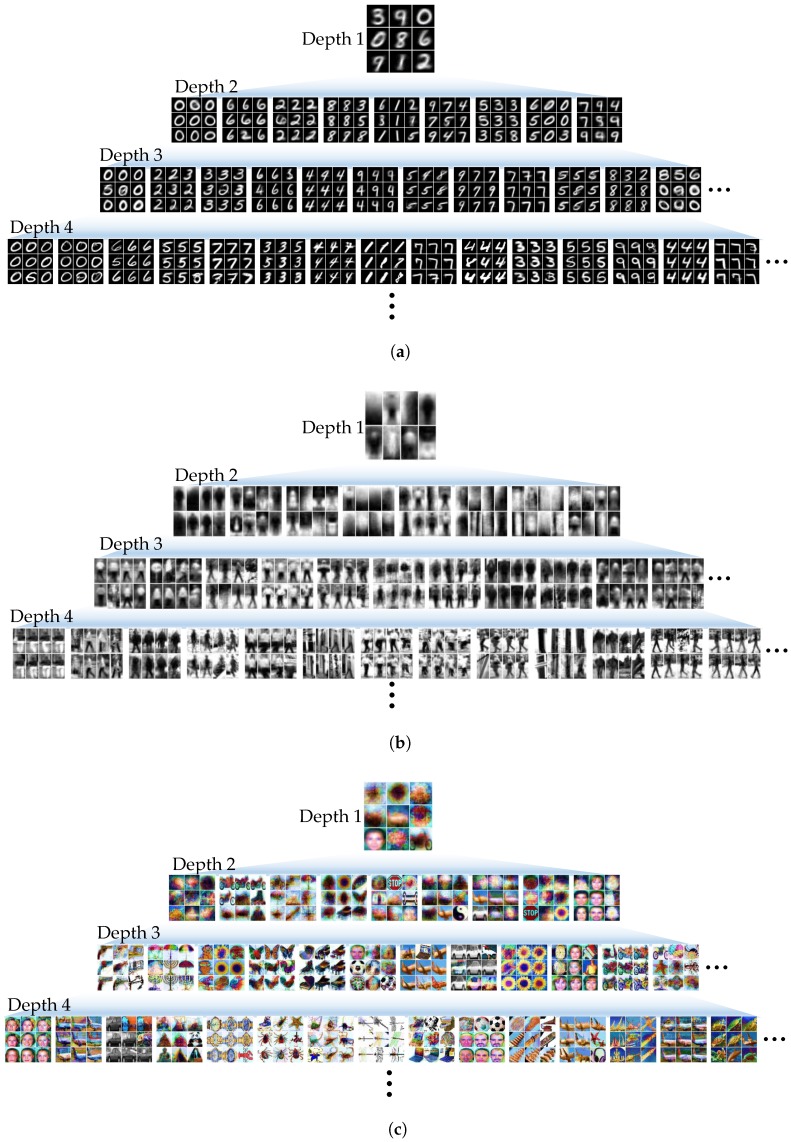
Visualization of node in one Opt-L-Tree with different depths on (**a**) MNIST dataset; (**b**) DMPB dataset; and (**c**) Caltech101 dataset

## Appendix A. Effect of Termination Condition and Ensemble Number

Generally, the specific thresholds for depth or number at last leaf node are utilized for termination conditions when the tree induced. These conditions could provide an advantage in increasing the generalization ability and preventing overfitting problems. However, it contains a possibility that traditional tree-induced methods do not have sufficient search for classification compared with the L-Tree-based method. To analyze the effect of the number of ensemble trees and the termination conditions, the MNIST dataset was employed by increasing the number of fully-developed trees (no predefined depth and minimum number samples at leaf node are 2) from 5 to 1000. As shown in Figure A1, the result represents the fully developed tree has better performance than the previous configuration. However, overall trends among the various tree methods except ERT were maintained. Since the ERT algorithm is able to learn more efficiently as the number of trees increases. However, it has the limitation to the performance under various conditions compared to the proposed L-Tree method because splitting criteria are highly dependent on the small amounts of pixels.
